# Axonal degeneration as a therapeutic target in the CNS

**DOI:** 10.1007/s00441-012-1362-3

**Published:** 2012-03-06

**Authors:** Paul Lingor, Jan C. Koch, Lars Tönges, Mathias Bähr

**Affiliations:** Department of Neurology, University Medicine Göttingen, Robert-Koch-Strasse 40, 37075 Göttingen, Germany

**Keywords:** Neurodegeneration, Neurotrauma, Wallerian degeneration, Calcium, Autophagy

## Abstract

Degeneration of the axon is an important step in the pathomechanism of traumatic, inflammatory and degenerative neurological diseases. Increasing evidence suggests that axonal degeneration occurs early in the course of these diseases and therefore represents a promising target for future therapeutic strategies. We review the evidence for axonal destruction from pathological findings and animal models with particular emphasis on neurodegenerative and neurotraumatic disorders. We discuss the basic morphological and temporal modalities of axonal degeneration (acute, chronic and focal axonal degeneration and Wallerian degeneration). Based on the mechanistic concepts, we then delineate in detail the major molecular mechanisms that underlie the degenerative cascade, such as calcium influx, axonal transport, protein aggregation and autophagy. We finally concentrate on putative therapeutic targets based on the mechanistic prerequisites.

## Introduction

The axon represents the largest functional entity in many neuronal populations, e.g. spanning up to more than one meter in human motoneurons. Whereas the dysfunction of the cell soma and its consecutive degeneration in the course of neurological disorders dominated the perception in neuropathology for many years, an increasing amount of data supports the concept of axonal degeneration as the initial pathological mechanism. Thus, it comes to no surprise that many approaches aiming at neuroprotection and mainly somatic mechanisms (not specifically targeting the axon) have failed in functional long-term trials and in humans. The consideration of axonal degeneration as a major mechanistic step is therefore mandatory for the design of successful therapeutic strategies in disorders of the central nervous system (CNS).

The discovery of mutant mice that show a delay in Wallerian degeneration after injury through expression of the WldS (slow Wallerian degeneration) mutant protein (Lunn et al. 1989) prompted the idea that axonal degeneration, similar to apoptosis, is a controlled mechanism that does not occur in a random fashion but can be regulated. In similarity to programmed cell death, conserved mechanisms seem to participate in the course of traumatic and degenerative axonal demise. An understanding of these molecular pathways should therefore not only enable us to interact and interfere with degeneration in a true neuroprotective approach, but also could lay grounds for regenerative attempts.

This review starts with an overview of the evidence for axonal degeneration in pathological conditions and then addresses the morphological and molecular processes of axonal destruction. Finally, we summarize putative therapeutic strategies based on the current knowledge of molecular mechanisms. Because of the wealth of data and the rapid growth of information on the topic, we have had to make a selection of cited works reflecting our effort to draw a global picture rather than to provide an encyclopaedic compilation. We therefore apologize to all colleagues whose work could not be included.

## Axonal degeneration in CNS disease

Traumatic injury to the brain or to the spinal cord most obviously involves primary axonal disruption. However, inflammatory disorders, such as multiple sclerosis (MS), and degenerative diseases, such as Alzheimer’s disease (AD) and Parkinson’s disease (PD), also show pronounced axonal pathology. We have to bear in mind that, although some parts of the degenerative cascade might indeed be similar in different pathologies, their origins may vary fundamentally. Membrane disruption in traumatic brain or spinal cord injury, for example, might eventually result in calcium influx, calpain-mediated cleavage and axonal transport breakdown. Axonal transport impairment, however, may also be caused by aggregation of proteins. Both membrane disruption and protein aggregation can thus converge on the very same common final path having a provenance in completely different pathologies. This illustrates that axonal degeneration cannot be regarded as a uniform sequence of events, but rather should be seen as a process with numerous variants, some of which share common key features.

In the following section, we discuss the neuropathological evidence with respect to axonal degeneration from traumatic, inflammatory and degenerative CNS disorders and their corresponding animal models.

### Traumatic CNS disorders

Traumatic spinal cord injury is a prime example of axonal damage that affects the longest projecting CNS neurons and peripheral afferents. Most cases of traumatic spinal cord injury result in a partial transection of ascending and/or descending tracts and incomplete impairment of sensory or motor functions, whereas only a small fraction represents complete transections (Rowland et al. 2008). Next to the initial axonal damage that occurs because of mechanical stress, secondary damage contributes to further dysfunction (Bramlett and Dietrich 2007). Primary damage to cortical neurons induces Wallerian degeneration of the distal axons projecting to the spinal cord. However, in addition to this direct lesion effect, neighbouring axons that have not been primarily affected show signs of degeneration after a lag period. This is accompanied by a local increase of Iba-1-positive microglia, which are hypothesized to be responsible for this secondary damage (Weishaupt et al. 2010).

The spinal cord lesion was also one of the first mammalian animal models in which axonal degeneration could be imaged in vivo. Selective labelling of dorsal root ganglion neurons by using transgenic mice expressing fluorophores in a subset of neurons allowed the visualization of degenerative changes within the first minutes after lesion and permitted the re-imaging of animals several days later. This pioneering technique permitted the identification of acute axonal degeneration (see Acute axonal degeneration) as a separate initial mechanism following traumatic lesions (Kerschensteiner et al. 2005).

Traumatic injury to the brain also results in pronounced axonal lesions, mostly attributable to shear stress (Meythaler et al. 2001). In contrast to focal spinal cord injury, axonal damage in traumatic brain injury is more diffuse. Complete transections are less frequent but the shear stress results in structural damage and bead formation in the axons, which eventually can lead to dysfunction and cell death (McIntosh et al. 1996).

Axonal membrane permeability appears to be rapidly altered after even moderate traumatic brain injury and this is accompanied by a local compaction of neurofilaments (Pettus et al. 1994). In a cat model of fluid percussion brain injury, these local permeability alterations are apparent as early as 5 min after trauma and the decreased spacing of neurofilaments persists at least for 6 h after the impact (Pettus and Povlishock 1996). Another commonly used model for traumatic brain injury is the stretch injury paradigm in cultured neurons in vitro. Following stretch, undulating distortions are formed along the axons; these are attributable to microtubule breakage and subsequent microtubule loss. Taxol treatment is able to attenuate consequent axonal degeneration, whereas nocodazole treatment promotes it. The downstream cascades of microtubule disruption are likely to involve axonal transport dysfunction (see Axonal transport) and protein accumulation (Tang-Schomer et al. 2010).

### Degenerative CNS disorders

#### Parkinson’s disease

Many neuropathological studies of PD brains now support the hypothesis that the loss of striatal dopaminergic terminals precedes the demise of dopaminergic neurons in the substantia nigra pars compacta, the demise having long been regarded as the major pathological hallmark of PD (for a review, see Burke 2010). Consistent with this idea, striatal dopamine levels decrease more strongly than the numbers of nigral dopaminergic neurons in PD patients (Kish et al. 1988). Remarkably, the first observable changes in early stage PD brains involve the appearance of Lewy neurites, which initially outnumber Lewy bodies localized in the dopaminergic cell soma (Braak et al. 2003). The axonal projections of the substantia nigra relay an inhibitory signal via dopaminergic transmission to the striatum. However, whereas the degeneration of this anatomical projection most prominently reflects the negative motor symptoms in the disease, it is indeed not the only fiber tract affected by degenerative pathology; as could be demonstrated by the studies of Braak and colleagues (2003), degenerative changes appear first in the dorsal motor nucleus of the vagal nerve, spreading further via the caudal raphe nuclei and nucleus coeruleus before reaching the midbrain. Synaptic dysfunction attributable to axonal pathology in the hippocampus and the amygdala of PD patients has been implicated in cognitive and emotional impairment in this disease (Bertrand et al. 2003). More recently, Orimo et al. (2008) have evaluated the degeneration of cardiac sympathetic nerves, which degenerate in PD and in Lewy body disease. Moreover, here, distal axonal degeneration precedes the loss of their mother neurons. Interestingly, alpha-synuclein aggregates are even more frequent in incidental Lewy body disease, in which tyrosine-hydroxylase-positive neurons are still preserved, than in PD patients, which further ferments the discussion of whether Lewy bodies *per se* are toxic or even protective in aggregopathies. Here, aggregates are predominantly found in the neuronal soma but the number of axons has already decreased markedly (Orimo et al. 2008). These observations in patients with idiopathic PD are similar to those in a brain of a familial PD patient carrying the alpha-synuclein (A30P) mutation (Seidel et al. 2010b). In addition to the degeneration of the nigrostriatal axonal projections, PD patients also show trans-synaptic degeneration, e.g. in the caudate nucleus. This has been made responsible for the poor clinical response of advanced stage PD patients to dopaminergic graft therapy (Lach et al. 1992).

This wealth of data suggests that, in PD, the initially occurring axonal pathology precedes neuronal cell death and that axonal degeneration is the histological substrate of clinically apparent motor and non-motor deficits.

Animal models based on genetic PD forms and toxin-based paradigms also suggest an involvement of axonal pathology in the beginning of the disease process. In a rat model of PD based on adeno-associated virus (AAV)-mediated expression of mutant human alpha-synuclein (aSyn.A53T) in the substantia nigra, neuronal loss occurs in a significant manner after 17 weeks. However, well before that, as early as 4 weeks after AAV-injection, dystrophic dopaminergic neurons in the striatum are found and a marked change occurs in proteins with functions in synaptic transmission and axonal transport (Chung et al. 2009). Axonal transport impairment (see Axonal transport) has also been observed in cultured neurons transfected with mutant forms of alpha-synuclein in vitro (Saha et al. 2004). Next to the overexpression of alpha-synuclein, the application of the neurotoxin 1-methyl-4-phenylpyridinium (MPP^+^) to primary dopaminergic neurons also leads to significant alterations of axonal transport mechanisms in vitro. Interestingly, MPP^+^ shows effects on mitochondrial movement, but not on synaptophysin-tagged vesicles or other moving particles, demonstrating that substrate specificity contributes to the particular toxicity of MPP^+^ in axons. In this model, neurite degeneration and an induction of autophagy (see Autophagy and the ubiquitin-proteasome system) also occur before cell body loss (Kim-Han et al. 2011).

Autosomal-dominant mutations in leucine-rich repeat kinase 2 (LRRK2) belong to the most frequent causes of familial PD. Large genome-wide association studies have identified LRRK2 in addition to alpha-synuclein as an important risk locus for the development of PD (Simón-Sánchez et al. 2009; Satake et al. 2009). Even the initial reports of LRRK2 function implied a role in neurite growth for this large multidomain protein (Macleod et al. 2006). In *Drosophila*, overexpression of the pathogenic LRRK2 (G2019S) mutant results in mislocalization of tau protein in dendrites and causes dendrite degeneration. The mechanism is suggested to involve an increased recruitment of the *Drosophila* glycogen synthase kinase 3 (GSK-3β) homolog (shaggy) by LRRK2 (G2019S), which in turn induces hyperphosphorylation and mislocalization of tau (Lin et al. 2010). Overexpression of the human mutant LRRK2 (R1441G) in a bacterial artificial chromosome transgenic mouse model supports the role of this protein in axonal stability. In addition to levodopa-sensitive motor symptoms, which have been observed at 10 months of age, these animals present fragmented dopaminergic axons, axonal spheroids and dystrophic neurites (Li et al. 2009). As can also be shown in SH-SY5Y cells in vitro, overexpression of mutant LRRK2 (G2019S) results in decreased neurite outgrowth and an increased number of autophagic vacuoles (see Autophagy and the ubiquitin-proteasome system) and this effect is mediated by mitogen-activated protein kinase (MAPK)/extracellular signal-regulated kinase (ERK) signalling (Plowey et al. 2008).

Models based on alpha-synuclein, LRRK2 or 1-methyl-4-phenyl-1,2,3,6-tetrahydropyridine (MPTP)/MPP^+^ appear substantially different at first glance and all these models only partially reproduce the pathophysiology of human PD. However, alterations in axonal transport, autophagy and an early axonal loss preceding cell death seem predominantly common themes that can be partially correlated to autopsy material suggesting an involvement of these processes in the human disease. Thus, the hope is raised that the targeting of any of these mechanisms in a therapeutic manner will modify the disease progression in humans independently of the underlying etiology.

#### Amyotrophic lateral sclerosis

Axonal degeneration in amyotrophic lateral sclerosis (ALS) has long been considered to occur only as a secondary process, the result of motoneuron apoptosis comparable to Wallerian degeneration (Przedborski 2004). Thus, the “dying-forward” hypothesis proposes that ALS is mainly a disorder of cortical motoneurons that connect monosynaptically with anterior horn cells and mediate anterograde degeneration of anterior horn cells via glutamate excitotoxicity. However, several animal studies have recently demonstrated that the exclusive protection of the neuronal cell body, e.g. through the regulation of pro- or anti-apoptotic proteins such as Bax or Bcl-2, does not prevent axonal degeneration or functional deterioration (Sagot et al. 1995; Gould et al. 2006). Even stronger evidence comes from human autopsy material. Fischer and colleagues (2004) have impressively demonstrated, in autopsy material from an ALS patient who died from an unrelated cause only 6 months after symptom onset, that denervation and reinnervation were pronounced, while the spinal motor neurons were not yet affected. Pathological examination of tissue from ALS autopsy cases and from transgenic mouse models of the disease demonstrates the appearance of swollen axonal segments or spheroids being rich in intermediate filament proteins including the neurofilament triplet proteins NFL, NFM and NFH (Hirano 1996). The pathophysiology that leads to the formation of these spheroids is most likely linked to axonal transport deficits (see Axonal transport) as has been shown in various mouse models (Rao and Nixon 2003). Recently, data from a cell culture model of axonopathy with spheroid formation, neurofilament and microtubule disorganization have underlined the important influence of the glial environment on the disruption of axonal transport (King et al. 2011). Hence, (1) the mechanisms of neuronal cell body demise must differ from axonal degeneration and the latter cannot be explained as a pure consequence of the somatic dysfunction and (2) axonal degeneration seems to precede neuron death in this disease, suggesting an additional “dying back” mechanism (for reviews, see Rowland and Shneider 2001; Fischer and Glass 2007).

This is also reflected in the SOD1 (G93A) mouse model of ALS, which is based on a mutation found in familial ALS cases and faithfully reproduces the clinical course of disease in humans (Gurney et al. 1994). Because of a clearly defined age at symptom onset, SOD1 (G93A) mice are an ideal model for the study of the presymptomatic alterations in this motoneuron disorder. Paralleling the findings from autopsy material, SOD1 (G93A) mice show a dramatic denervation of motor end plates well before the onset of clinical disease; whereas motor weakness starts to be apparent only at around 80 days, about 40% of the neuromuscular junctions show denervation. However, at this time, no evidence for motoneuron cell loss is detectable (Fischer et al. 2004). The situation seems to be slightly different for corticospinal motoneurons. In the same SOD1 (G93A) model, early and cell-type specific apoptosis has been observed among neocortical neurons. Here, the degeneration of the corticospinal tract appears to follow neuronal demise, which can be visualized by using double-transgenic Thy1-YFP/SOD1 (G93A) mutants (Ozdinler et al. 2011). A recent study by Bilsland et al. (2010) clearly demonstrates that axonal transport deficits occur in presymptomatic mice and represent the earliest axonal pathologies in this model, occurring well before motoneuron loss.

In conclusion, data from ALS and motoneuron disease models suggests that axonal degeneration probably precedes motoneuron death. This obviously does not exclude the presence and negative additional impact of somatic dysfunction on disease progression.

#### Alzheimer’s disease

One of the prominent neuronal structural alterations in AD, other than amyloid plaques and neurofibrillary tangles, are dystrophic neurites (Onorato et al. 1989). These neurites are characterized by the disorganization of the microtubule and the neurofilament network (Boutajangout et al. 2004) and the abnormal accumulation of variably phosphorylated neurofilaments that can be found even in early stages of the disease (Dickson et al. 1999). A recent study in human patients with mild cognitive impairment and early AD has visualized functionally relevant alterations of the axonal tracts in the white matter of the brain by employing diffusion tensor imaging, thereby confirming that axonal degeneration is a measurable pathological feature even at early stages of this disease in humans (Huang et al. 2007).

Moreover, a number of animal models for AD have been developed and extensively studied as a surrogate for neuropathological alterations observed in human patients. Pronounced axonal degeneration has been reported for most of these models, including models with underlying mutations of tau (Probst et al. 2001), ApoE (Tesseur et al. 2000), and amyloid precursor protein (APP; Wirths et al. 2007), to name only a few examples. These axonal defects are typically characterized by large axonal swellings, often in close proximity to the amyloid plaques (Stokin et al. 2005). For example, in 5XFAD mice expressing a triple-mutated human APP, prominent axonal swellings are found in the brain and spinal cord even at 3 months of age (Jawhar et al. 2010). Many dystrophic neurites can be seen close to extracellular plaques. The motor deficits observed in these mice correlate with the formation of axonal spheroids in the brain and spinal cord (Oakley et al. 2006; Jawhar et al. 2010). The interaction of extracellular plaques and the intra-axonal milieu is a matter of intense current research and a better understanding of this interaction might shed light on the function of protein aggregates for axonal degeneration in a number of aggregopathies.

### Chronic inflammatory CNS disorders

For decades MS, as the prime example for chronic inflammatory CNS disorders, has been regarded as a largely demyelinating disorder. However, studies of brain tissue from MS patients have changed this picture completely; axonal transections with morphological changes resembling traumatic axonal injury, such as axonal ovoids, are abundantly present in acute and chronic lesions (Ferguson et al. 1997; Trapp et al. 1998). Characteristics of Wallerian degeneration can be identified in the periplaque white matter in early MS, reaffirming the finding that axonal lesions represent the morphological correlate of the persistent neurological deficits in MS patients (Dziedzic et al. 2010).

Animal models of MS, such as the experimental autoimmune encephalomyelitis (EAE) model induced by immunization with myelin oligodendrocyte glycoprotein, show axonal degeneration as an important feature and this can even precede the demyelinating component (D. Wang et al. 2005). Using multiphoton in vivo imaging techniques, Nikić et al. (2011) have been able to demonstrate that focal axonal degeneration (FAD, see Chronic axonal degeneration) might precede demyelination in a mouse model of MS. Focal intra-axonal mitochondrial alterations can be observed, even before the ultrastructural signs of axonal damage. In this model, reactive oxygen and nitrogen species, which are likely to be derived from activated macrophages, are able to initiate mitochondrial damage and FAD. Most intriguingly, the authors have been able to show that these initial axonal alterations can be reversible up to a certain time point, opening a novel window for intervention. Similar changes have also been observed in human biopsy tissue of MS patients underlining the importance of these findings (Nikić et al. 2011).

## Mechanisms of axonal degeneration

In this section, we will first describe basic concepts of axonal demise; these concepts are initially described based on their morphological and temporal characteristics. Secondly, we will take a closer look at the molecular mechanisms underlying axonal damage, thereby making clear that such mechanisms can represent underlying events of morphologically different degenerative processes.

### Mechanistic concepts

Like apoptosis, most forms of axonal degeneration seem to be active self-destructing cellular processes involving a determined cascade of various molecular players (Raff et al. 2002). We need however to note that apoptosis and axonal degeneration employ independent biochemical pathways and can be initiated and modulated separately (Whitmore et al. 2003). Different forms of axonal degeneration have been described with regards to localization on the axon and time kinetics. Although common molecular convergence points probably exist, important mechanistical differences are found between each type of axonal degeneration. The most extensively studied form of axonal degeneration is the sequential degeneration following a traumatic lesion of an axon. This includes acute axonal degeneration in the vicinity of the lesion and Wallerian degeneration of the distal part of the axon. The study of axonal degeneration in chronic neurological diseases represents a greater challenge as this process does not occur simultaneously in all axons of a certain tract and often proceeds over extended time periods. Nevertheless, FAD, axonal die-back and Wallerian degeneration have been described for these conditions.

#### Acute axonal degeneration

**Fig. 1:**
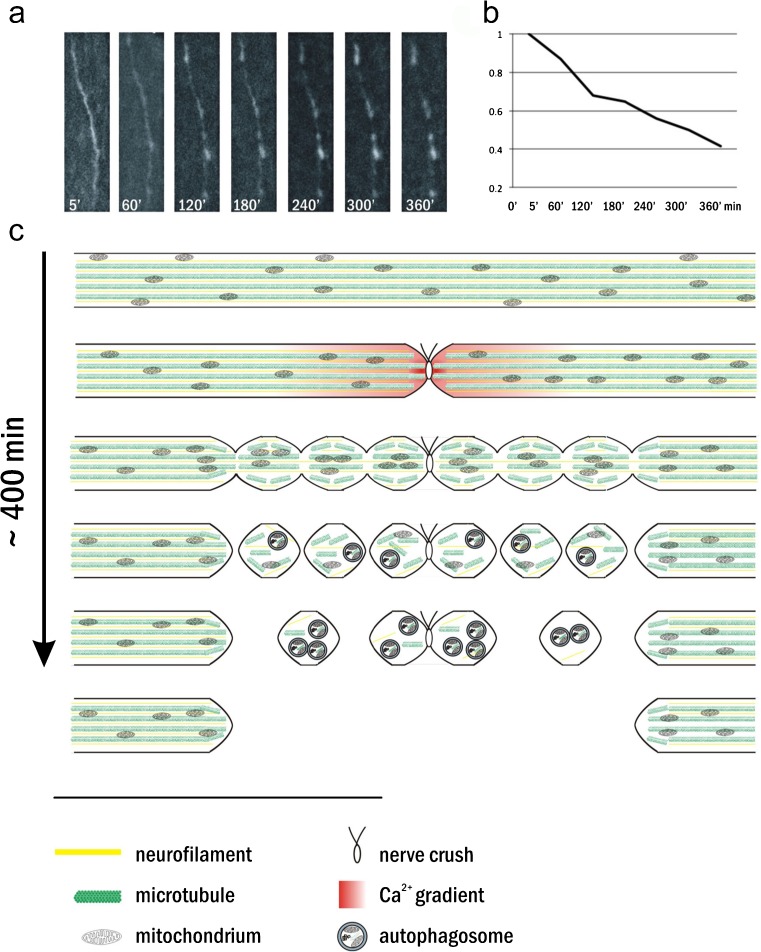
**a** Micrographs of a rat optic nerve axon labelled with enhanced green fluorescent protein expressed by an intravitreally injected viral vector at given time points (in min) after optic nerve crush (proximal to the crush site). **b** Quantification of the axonal integrity ratio (sum length of the remaining axonal fragments divided by initial length of the intact axon segment) of the axon in **a**. **c** Representation of the morphological events observed in acute axonal degeneration. A crush lesion of an axon of the central nervous system leads to a rapid increase of intracellular calcium concentrations within the first 30-40 s after lesion. Misalignment of neurofilaments and disruption of microtubules followed by local accumulations of organelles attributable to dysfunctional axonal transport and the subsequent formation of axonal bulbs can be seen within the next 30-120 min. This is then followed by the appearance of a high number of autophagic vacuoles and the fragmentation of the axon spanning over 400 μm proximal and distal from the crush site

The term acute axonal degeneration refers to a rapid axonal disintegration within several hours following a traumatic lesion in the CNS. It is confined to the adjacent 300–400 μm of the proximal and distal end of the axon and has been described for the spinal cord (Kerschensteiner et al. 2005) and the optic nerve (Knöferle et al. 2010). In both cases, it has been visualized by using in vivo live-imaging techniques. Although the time kinetics differ slightly between the model systems, being faster in the mouse spinal cord than in the rat optic nerve, the sequential morphological changes and the undelying mechanisms seem to be similar in both tissues (summarized in Fig.1).

At first, for 10–30 min after a traumatic lesion, the axon remains completely stable with regard to its macroscopic morphological appearance. On the molecular level, however, a signalling cascade has already been activated, finally resulting in the fragmentation of the axon. It is initiated by a rapid calcium influx into the axon and a consecutive transient rise of the axoplasmic calcium concentration within 40 s after lesion (see Calcium). Application of calcium channel inhibitors at the time of the lesion blocks this rise in cytosolic calcium and almost completely inhibits acute axonal degeneration (Knöferle et al. 2010). The calcium influx leads to an activation of the calcium-sensitive protease calpain, which reaches its maximum 30 min after the lesion. Local application of calpain inhibitors can completely inhibit consecutive acute axonal degeneration (Kerschensteiner et al. 2005). Calpain regulates a multitude of neuronal substrates (for a review, see Vosler et al. 2008) but which of these are most relevant for axonal degeneration is currently still under investigation (see Calcium-triggered events).

The first changes at the ultrastructural level become visible within the first 30 min after lesion and consist of the condensation and misalignment of neurofilaments followed by the fragmentation of microtubules (Knöferle et al. 2010). Both focal neurofilament compaction and microtubular proteolysis have been linked to calpain activation in other disease models in the CNS, and the ERK/MAPK pathways have been suggested as molecular mediators (Pettigrew et al. 1996; Veeranna et al. 2004). Therefore, the initial calpain activation might also be responsible for these early ultrastructural changes in acute axonal degeneration. Moreover, the rapid breakdown of the cytoskeleton probably leads to the early impairment of axonal transport. Indirect signs in favour of this assumption are accumulations of organelles, mainly mitochondria and vacuoles, eventually leading to local axonal swellings that can be found early in axons undergoing acute axonal degeneration (Knöferle et al. 2010).

Another characteristic ultrastructural feature of acute axonal degeneration is a local activation of autophagy (see Autophagy and the ubiquitin-proteasome system). The number of autophagosomes in the axon significantly increases within the first 6 h after lesion. Pharmacological inhibition of autophagy markedly attenuates acute axonal degeneration but this effect, however, is not as pronounced as after calcium channel blockage. The latter not only inhibits acute axonal degeneration, but also reduces autophagy, suggesting that autophagy is a downstream target of calcium influx (Knöferle et al. 2010; Koch et al. 2010).

Whereas pharmacological interventions such as the inhibition of calcium, calpain and autophagy are highly effective in preventing acute axonal degeneration, the long-term effects of these treatments are still unclear. As acute axonal degeneration only affects ∼400 μm of the axon proximal to the lesion site, the direct functional benefit of rescuing this short portion of the axon is negligible. Nevertheless, one can argue that the regenerative response of a lesioned axon might be fostered as the distance to the rapidly forming, growth-inhibiting scar tissue becomes smaller. Kerschensteiner et al. (2005) have shown that regenerating sprouts can be seen within 24 h after lesion. Although these early axonal sprouts lack appropriate directional information, they could possibly benefit from an inhibited acute axonal degeneration; this however remains to be experimentally established.

#### Wallerian degeneration

Wallerian degeneration classically refers to the degeneration of axons distal to a lesion site (Waller 1850). After a traumatic lesion, the parts of the axon that are not affected by acute axonal degeneration initially stay morphologically stable for the first 24 to 72 h. Then, the distal part of the axon undergoes a progressive fragmentation that resembles the fragmentation seen in acute axonal degeneration (Kerschensteiner et al. 2005) and that finally leads to a complete removal of the distal part of the axon. Wallerian degeneration proceeds directionally along the axon with a speed ranging from 0.4 mm/h in cultured primary neurons (Sievers et al. 2003) to 24 mm/h in the mouse sciatic nerve (Beirowski et al. 2005). In the peripheral nervous system, the direction of Wallerian degeneration on the axon seems to depend on the lesion type; complete transection of the nerve leads to an anterograde fragmentation proceeding from proximal to distal, whereas a crush lesion results in retrograde fragmentation starting at the far distal end of the axon (Beirowski et al. 2005). Although macrophages and glia play an important role, especially in the final removal of the axon fragments, the mechanism of Wallerian degeneration seems to be intrinsic to the axon (Glass et al. 1993; Hoopfer et al. 2006; MacDonald et al. 2006).

The molecular machinery underlying Wallerian degeneration is still not completely understood, although great progress has been made with the help of the so-called WldS mouse (Lunn et al. 1989). In this mouse mutant, axon stumps distal to the lesion site survive ten times longer than axons in wild-type animals, while the survival of the neuronal cell body is not altered (Deckwerth and Johnson 1994; Adalbert et al. 2006).

The mutant protein WldS, which is responsible for slowing down the degenerative process in WldS mice, is a chimeric gene product consisting of a fragment of the polyubiquitination factor UFD2a/UBE4b and the full-length nicotinamide mononucleotide adenylyltransferase-1 (NMNAT1; Mack et al. 2001). NMNAT1 is a key protein of the nicotinamide-adenine dinucleotide^+^ (NAD^+^) salvage pathway in mammals. UBE4b is an E4-type ubiquitin ligase that can add multiubiquitin chains to substrates of the ubiquitin/proteasome degradation pathway (Hatakeyama et al. 2001). The functionally most important molecular sites of WldS are the ATP-binding site and the NMN^+^ binding site of NMNAT1 and the valosin-containing protein (VCP)-binding site of UBE4b, as has been shown by knock-out experiments. Both a functional NMNAT1 and a functional UBE4b fragment seem to be required for the neuroprotective action of WldS. This is suggested by the observation that, although disruption of the enzymatic activity of NMNAT1 in transgenic WldS mice results in a strongly reduced neuroprotective phenotype (Avery et al. 2009; Conforti et al. 2009; Yahata et al. 2009), the overexpression of NMNAT1 alone is not sufficient to protect lesioned axons from degeneration in mammalian neurons (Conforti et al. 2009). Moreover, NMNAT-1 has been demonstrated to function not only with enzymatic activity, but also as a chaperone, at least in biochemical assays and cultured cells (Zhai et al. 2003).

The WldS protein is located mainly in the nucleus but has also been detected in the axoplasm and axoplasmic organelles (Beirowski et al. 2009; Yahata et al. 2009). Local overexpression of NMNAT1 targeted to the axonal compartment results in protective effects resembling those of the WldS transgene (Beirowski et al. 2009; Sasaki et al. 2009a; Babetto et al. 2010). These data suggest that the protective action of WldS is mediated by the continuous transport of the protein along the axon. In agreement with this, other NMNAT isoforms have been shown to promote axonal survival locally. NMNAT2 is continuously transported from the cell body to the axon and has an extremely short turnover time of ∼4 h (Gilley and Coleman 2010). Short interfering RNA (siRNA)-mediated downregulation of NMAT2 or inhibition of its transport to the axon lead to the induction of axonal degeneration, whereas overexpression delays axotomy-induced degeneration (Gilley and Coleman 2010; Yan et al. 2010). Similar positive effects on axonal survival and integrity can be achieved by the overexpression of the mitochondrial isoform NMNAT3 (Avery et al. 2009; Yahata et al. 2009). The downstream targets of the NMNAT isoforms for promoting axonal survival still remain elusive. All NMNATs contain a catalytic domain for the synthesis of NAD^+^ (Berger et al. 2005). The data concerning the role of NAD^+^ in the maintenance of axonal survival are however inconsistent. Extracellular application of high concentrations of NAD^+^ result in the protection of injured axons in vitro, whereas various attempts to increase the intracellular NAD^+^ concentration have not had an effect on axonal degeneration (Sasaki et al. 2009b). The local action of NAD^+^ in subcellular departments within the axon involving local energy supply (J. Wang et al. 2005), the local sequestering of Ca ions or the modulation of ion channel activity (Tamsett et al. 2009) have been argued to account for NAD^+^-mediated effects in the axon but these hypotheses still need to be established by experimental evidence.

#### Chronic axonal degeneration

The time kinetics of axonal degeneration in chronic neurodegenerative diseases are more challenging to study. However, various morphological forms of axonal degeneration have been described for these conditions that may also occur in parallel.

The first type is termed “dying back degeneration” (Cavanagh 1964). This form of degeneration has been described in ALS (Sobue et al. 1983; Nihei et al. 1993), diffuse Lewy body disease (Iseki et al. 1998), spinocerebellar ataxia (Seidel et al. 2010a), peripheral neuropathies (Vavlitou et al. 2010) and toxic neuropathies (Bennett et al. 2011), amongst others. It is initiated by a dysfunction of the synaptic connection and/or a degeneration of the distal regions of the axon. This is then followed by a degeneration of the whole axon in a distal-to-proximal direction, finally leading to a fragmentation of the axon morphologically resembling that of Wallerian degeneration (Cavanagh 1979). The biochemical mechanisms underlying this form of degeneration are currently not completely clear but synaptic pathology (Chang et al. 2006), mitochondrial dysfunction (Shi et al. 2010) and disturbances of axonal transport (Morfini et al. 2007; Bilsland et al. 2010) have been implied.

Dying back degeneration shares striking similarities with axonal pruning and axosome shedding, a process that is observed for example in the developmental maturation of the neuromuscular synapse or target selection, e.g. by retinal ganglion cell axons (for excellent reviews, see Luo and O’Leary 2005; Misgeld 2011).

Another form of axonal degeneration has been recently visualized in a model of chronic inflammatory disease: FAD. Morphologically, a focal swelling of the axon is observed at the beginning and is characterized by an accumulation of organelles and dysmorphic mitochondria. This focal swelling is accompanied by a dysfunction of axonal transport and eventually leads to a local disruption of the axon followed by a Wallerian-like fragmentation of the axon stumps. FAD has recently been imaged in vivo in an animal model of MS (Nikić et al. 2011). In this EAE model, increased levels of reactive oxygen species in the inflammatory EAE lesions are able to induce focal axonal swellings and these have been shown to be partially reversible (see Chronic inflammatory CNS disorders).

### The underlying molecular machinery

**Fig. 2:**
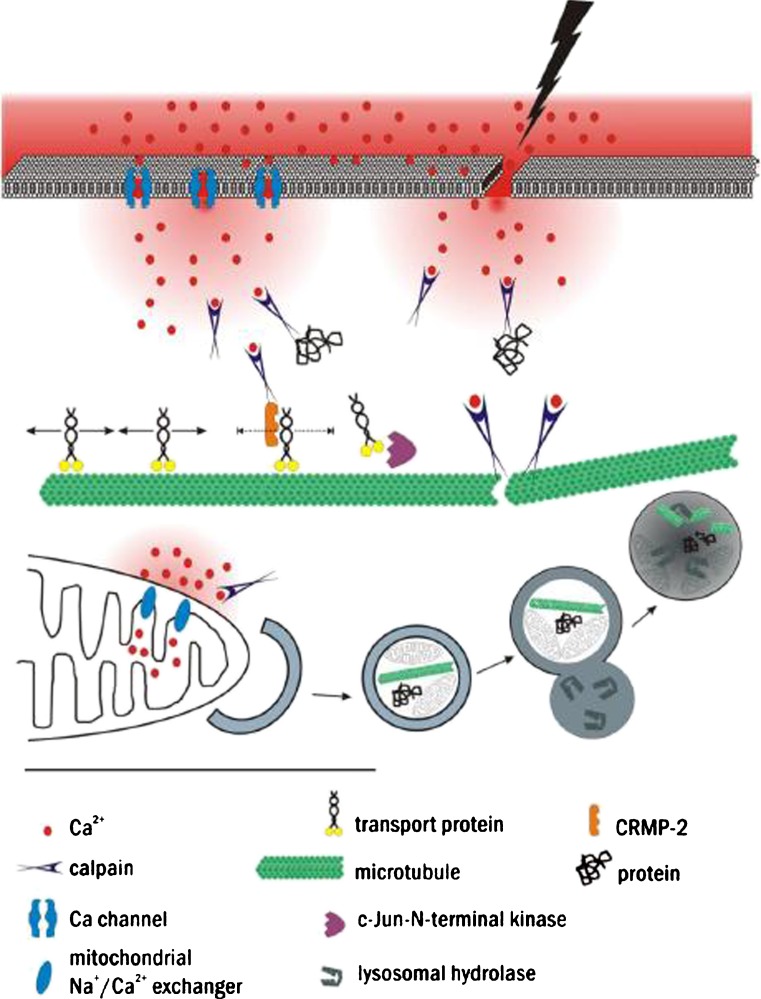
Representation of selected molecular mechanisms involved in axonal degeneration. Intra-axonal calcium levels can rise by the entry of calcium from the extra-axonal space, e.g. through calcium channels and disrupted axonal membranes, and from intra-axonal sources, e.g. mitochondria or endoplasmic reticulum (not shown). Calcium-dependent proteins, such as calpain, are activated and cleave target proteins, e.g. microtubules or collapsin response mediator protein-2 (*CRMP-2*). This in turn can result in axonal transport impairment. Autophagy is induced in a calcium-dependent manner and results in the degradation of proteins and organelles

Acute axonal degeneration, focal axonal degeneration and Wallerian degeneration appear to be specific morphological expressions of a cumulative number of underlying molecular mechanisms. We review here some of the most important and so far best-characterized molecular mechanisms that form the backbone of the degenerative cascade (summarized in Fig. 2).

#### Calcium

Several means have been experimentally shown to contribute to the elevation of axoplasmic calcium concentration in various lesion paradigms: (1) calcium influx from the extracellular space through disrupted membranes, (2) calcium-channel-mediated calcium influx from the extracellular space and (3) release of calcium from intracellular calcium stores (for a comprehensive review, see Stirling and Stys 2010).

Mechanical axonal lesions result in the disruption of the membrane continuity and subsequent influx of extracellular calcium into the cytoplasm. As Ziv and Spira (1995) have demonstrated using calcium-sensitive dyes in cultured *Aplysia* axons in vitro, the calcium wave induced through the transection of one neurite branch rapidly spreads, within seconds, until the next branching point where it seems to be halted. Intra-axonal calcium concentrations decrease subsequently within several minutes reaching initial levels after the resealing of the lesioned axon end. However, intracellular calcium concentrations reach a maximum of up to >1 mM near the lesion site and of up to several hundred micromolar more distal from it (Ziv and Spira 1995). Axotomy-induced calcium increase in vitro has been shown to require extra-axonal calcium levels of >200 μM and calcium has been demonstrated to enter axons via L-type, but not N-type calcium channels in dorsal root ganglion axons (George et al. 1995). Studies of the rat optic nerve however have suggested the additional involvement of other calcium channels (P/Q-type, N-type) in the mediation of calcium influx into the lesioned axon (Fern et al. 1995; Alix et al. 2008), suggesting that the results depend on the examined region of the nervous system.

The extra-axonal space appears to be not the only source of calcium, as has been demonstrated by studies employing a calcium-depleted extracellular medium. Here, axonal damage can equally elicit an intra-axonal calcium increase demonstrating that intracellular calcium stores can also contribute to cytoplasmic calcium release. For example, in ischaemic damage of dorsal column axons, calcium has been shown to be released from the endoplasmic reticulum through ryanodine receptors (Ouardouz et al. 2003) or from mitochondria (Nikolaeva et al. 2005).

In myelinated axons, ryanodine receptors can be activated through L-type Ca channels, similar to so-called excitation-contraction coupling in skeletal muscle cells. Both ryanodine receptors and L-type Ca channels seem to be colocalized near the axolemma of myelinated axons. However, the blockage of intra-axonal calcium release, e.g. through the L-type calcium channel blocker nimodipine, only partially protects axons from degeneration suggesting that other sources of calcium or other non-calcium-dependent mechanisms contribute to axonal degeneration (Ouardouz et al. 2003). For example, intra-axonal calcium levels are known to increase when an ischaemia- or hypoxia-induced lack of energy supply results in the breakdown of the Na^+^/Ca^2+^ exchanger (Stys et al. 1992). In ischaemia, the majority of the intra-axonal calcium release appears to be mediated through the Na^+^/Ca^2+^ exchanger as can be demonstrated in experiments with CGP37157, a blocker of the Na^+^/Ca^2+^ exchanger in mitochondria (Nikolaeva et al. 2005). In this model, the application of cyclosporine A, a blocker of the mitochondrial permeability transition pore (mPTP), does not provide sufficient protection, thereby arguing against mPTP involvement. However, as Barrientos et al. (2011) have recently demonstrated in another model, the last word on the role of mPTP in axonal demise has not yet been spoken; axons in optic nerve explants show less mitochondrial swelling after the application of cyclosporine A and are indeed protected from degeneration. This effect has been further pinned down to the function of cyclophilin D (CypD), a component of the mPTP in neurons, the axons of which are protected after siRNA-mediated depletion of CypD (Barrientos et al. 2011).

Finally, excess calcium in neurons can be extruded by membrane-located pumps, such as the plasma-membrane calcium ATPase isoform 2. A decrease in the levels of this pump can promote axonal pathology in animal models of MS (EAE) and spinal cord injury (Nicot et al. 2003; Kurnellas et al. 2005).

#### Calcium-triggered events

Calcium influx is followed by the activation of calcium-dependent proteases, such as calpains, which cleave and degrade cytoplasmic proteins. An increased activity of calpains has been demonstrated for diffuse axonal injury following brain trauma and also in stroke, spinal cord injury and neurodegenerative diseases (Kampfl et al. 1996; Vosler et al. 2008). Calpains can proteolytically degrade a plethora of targets, such as cytoskeletal proteins, enzymes, receptors, channels and transcription factors (for a review, see Saatman et al. 2010). As such, calpains have also been shown to degrade substrates of crucial importance to axonal stability. Neurite degeneration as a result of growth factor deprivation (e.g. of nerve growth factor) also leads to calpain activation. This is followed by proteolytic cleavage of the collapsin response mediator protein-2 (CRMP-2; Touma et al. 2007). Because CRMP-2 binds to kinesin-1 and participates in axonal transport, cleavage by calpain interferes with cargo delivery (see Aggregation under Therapeutic targets).

In addition to calpain, other calcium-dependent enzymes are known to participate in axonal degeneration. For example, transient axonal stretch injury in vitro results in calcium release primarily from intracellular stores and this is followed by a delayed intracellular calcium level rise over 48 h. Here, the inhibition of the calcium-dependent phosphatase calcineurin attenuates secondary axonal degeneration (Staal et al. 2010). Calpain and calcineurin are only two examples of calcium-dependent proteins that act further downstream and that have been studied in more detail in this context.

#### Mitochondrial damage

Mitochondria appear to take a key role in the initial localization of axonal continuity disruption. In models of traumatic brain injury in which diffuse axonal injury is a major pathophysiological component, calcium influx is thought to be mediated through axolemmal pores. However, axonal damage and calpain activation does not occur simultaneously over the entire length of the axon but shows focal peaks colocalizing with mitochondria (Kilinc et al. 2009). This focal accumulation of mitochondria might be attributable to focal disruption of the cytoskeleton and cargo accumulation but could equally contribute to a further focal increase in calcium levels from accumulating mitochondria (see Calcium).

In FAD, mitochondria are damaged by reactive oxygen and nitrogen species, which are likely to be derived from macrophages, and this again triggers further axonal degeneration (Nikić et al. 2011). As mentioned earlier (see Calcium), the activation of the mPTP seems to play a pivotal role in triggering axonal destruction in axons of the CNS and peripheral nervous system (PNS) axons (Barrientos et al. 2011). In a model of familial ALS, mitochondria seem to participate in apoptotic cell death and consequent axonal degeneration, because the deletion of two neuronal pro-apoptotic Bcl-2 family members, Bax and Bak, is able to prevent axonal degeneration (Reyes et al. 2010). The debate on the relevance of mitochondrial motility is not completely settled as yet, since reports have also demonstrated that increased mitochondrial transport, as observed in syntaphilin knock-out mice, does not support survival in the SOD1 (G93A) mouse model (Zhu and Sheng 2011). On the other hand, motility impairment has been shown in a model of Huntington’s disease in which mutant huntingtin interacts with the mitochondrial protein Drp1 and results in defective mitochondrial movement and synaptic deficiencies (Shirendeb et al. 2011). Amyloid-beta (Aβ) has recently also been shown to impair mitochondrial motility and neurons treated with Aβ have shorter mitochondria, a feature that eventually results in synaptic degeneration (Calkins and Reddy 2011). Even more evidence comes from a histological study in brain samples of MS patients with chronic and active lesions in which alterations in mitochondrial density and function have been detected. Here, the activity of the mitochondrial respiratory chain complex IV has been shown to be reduced in demyelinated axons at the active edge of chronic active lesions but, interestingly, in chronic lesions, complex IV activity and mitochondrial mass are increased, possibly because of adaptive processes occurring during disease progression (Mahad et al. 2009).

The above-mentioned findings are only a few examples underlining that alteration of mitochondrial function, mitochondrial trafficking and activation of the mitochondrial apoptotic pathway, all of which might contribute to axonal damage in the different contexts of CNS pathology. A local lack of energy supply attributable to mitochondrial failure and a local increase of calcium concentrations might both be crucial factors.

#### Aggregation

Amyloidogenic proteins, such as alpha-synuclein, tau and Aβ, have been suggested to promote axonal degeneration in several neurodegenerative disorders, e.g. through interference with axonal transport mechanisms. Of course, the aggregation of proteins does not occur in all paradigms of axonal degeneration but it may promote axonal pathology in aggregopathies. For example, the overexpression of human wild-type alpha-synuclein by lentiviral vectors has recently been shown to result in aggregation and a clear degenerative phenotype in CNS axons (Decressac et al. 2011). Aggregation of amyloidogenic proteins cannot be regarded as a stand-alone phenomenon, since recent data have demonstrated that increased calcium concentrations accelerate alpha-synuclein aggregation in solution and in cultured cells (Nath et al. 2011). Thus, we can hypothesize that axonal lesions with consequent calcium influx also drives the aggregation of amyloidogenic proteins. Vice-versa, the presence of alpha-synuclein itself appears to influence trauma-induced axonal degeneration. Transgenic mice overexpressing the human alpha-synuclein (Thy1-αSyn_WT_) possess alpha-synuclein aggregates in the axons of the sciatic nerve. These animals display increased Wallerian degeneration after axotomy of the sciatic nerve, whereas in mice without alpha-synuclein expression (C57BL/6-Ola-hsd strain by Harlan B6), axonal degeneration occurs at a significantly slower rate (Siebert et al. 2010). This is intriguing, because alpha-synuclein is predominantly attributed a function in neurodegeneration of the CNS. This study, however, suggests that alpha-synuclein is involved in a more general mechanism of axonal destruction, which is also important for the PNS and traumatic lesions, and might be independent of a chronic neurodegenerative process per se. Its precise mode of action is as yet unresolved but accumulating data have led to the proposal of a direct interaction with the cytoskeleton; the colocalization of alpha-synuclein has been reported not only with cytoskeletal proteins, such as neurofilaments, tau and tubulin (Jensen et al. 1999; Alim et al. 2002; Kanazawa et al. 2008), but also for transport proteins, such as dynein and kinesin-1 (Utton et al. 2005).

Axonal inclusions have also been found in brains of patients with spinocerebellar ataxia type-3 (SCA3). Ubiquitin-positive aggregates have been observed in axonal projections that are known to degenerate in SCA3 patients. It is likely that these aggregates are detrimental to axonal transport mechanisms; this however remains to be established (Seidel et al. 2010a). Similar evidence comes from animal models of Huntington’s disease. Here, axonal accumulations of aggregated huntingtin result in transport defects that precede neuronal cell death (Li et al. 2001).

In AD, Aβ has been identified as one of the main mediators of axonal degeneration (Yankner et al. 1990; Emre et al. 1992). In vitro, the exposure of cultured rat sympathetic neurons to Aβ induces axonal degeneration, which only in the second place triggers the activation of caspases and subsequent neuronal cell death. This observation supports the hypothesis of primary axonal degeneration as being an important step in the pathogenesis of AD. In vivo, the clear spatial association of axonal swellings and dystrophic neurites with amyloid plaques points to a causal relationship between Aβ and axonal degeneration (Stokin et al. 2005). Transcranial two-photon imaging in vivo has been able to visualize dendritic damage by amyloid deposits near neurites; such damage leads to spine loss, shaft atrophy, varicosity formation and finally continuity disruption (Tsai et al. 2004). This is further strengthened by studies demonstrating that injections of Aβ in brains of normal rats can induce neurodegeneration (Kowall et al. 1991; Frautschy et al. 1991). However, this effect seems to be dependent on the aggregation state of Aβ as some forms of Aβ do not cause neurodegeneration, whereas the fibrillar form seems to be most neurotoxic (Podlisny et al. 1993; Snow et al. 1994). Interestingly, Aβ-mediated axonal toxicity can be rescued by calpain inhibition, which in turn also prevents apoptosis, suggesting a close interplay of amyloidogenic proteins and calcium metabolism (Song et al. 2006).

#### Axonal transport

If we assume that axonal degeneration in traumatic or degenerative disease is a mechanistically defined process, then plausibly the disruption of a strategically important entity, such as axonal transport, will ultimately result in malfunction. Because of their enormous length in relation to the cell soma, axons rely on an efficient transport system to target proteins and organelles to specific compartments, such as synaptic terminals or nodes of Ranvier. Deficiencies in axonal transport have been visualized in various models mimicking neurodegenerative disorders, such as motoneuron disease, PD, Alzheimer’s dementia, Huntington’s disease and Charcot-Marie-Tooth disease (for reviews, see De Vos et al. 2008; Morfini et al. 2009).

Axonal transport is mediated by two major groups of proteins: the kinesins, which mediate anterograde transport, and the dyneins, which are responsible for the retrograde counterpart. Mice expressing the mutant kinesin superfamily member KIF1Bβ show transport defects for synaptic vesicles and progressive muscle weakness attributable to a peripheral neuropathy. In humans, mutations in the *KIF1B* gene have been found in patients with a hereditary polyneuropathy (Charcot-Marie-Tooth disease, type 2A; Zhao et al. 2001). Transport defects induced by mutations in kinesin light-chain-1 can also activate stress kinases, such as c-Jun-N-terminal kinase (see Kinase activation), and this again can result in abnormal phosphorylation and aggregation of tau (Falzone et al. 2009). Expectedly, retrograde transport defects are no less relevant; missense point mutations in the dynein heavy-chain induce motoneuron degeneration in heterozygous mice and, in homozygous animals, the formation of inclusion bodies has been observed (Hafezparast 2003). Dynein mutations might also be a link to axonal degeneration in motoneuron disease, as a recent study nicely connects axonal transport deficits to autophagic impairment and subsequent clearance deficits for aggregated proteins (Ravikumar et al. 2005).

Deficits in axonal transport are also key pathophysiological features in AD and several publications support the hypothesis that the disturbed axonal transport in AD is either directly or indirectly caused by Aβ (see also Alzheimer’s disease and Aggregation above). For example, accumulations of APP as an indicator of disturbed axonal transport have been found in a subset of axons in TgCRND8/YFP-H mice and alterations in mitochondrial transport have been visualized in TgCRND8/YFP-H/Mito triple transgenics. However, the morphology of the mitochondria suggests rather a partial block than a complete breakdown, which might be correlated with the chronic development of pathology in this model (Adalbert et al. 2009). In another study, intracellular oligomeric Aβ inhibits bidirectional axonal transport via the activation of endogenous casein kinase 2 (Pigino et al. 2009). Application of pharmacological inhibitors of casein kinase 2 is able to prevent the impairment of axonal transport by oligomeric Aβ. The presence of transport deficits has been also demonstrated in vivo, manganese-enhanced magnetic resonance imaging (MRI) being used in the Tg2576 mouse model of AD to assess axonal transport. In young mice, before Aβ deposition, axonal transport rates have been shown to be normal. After the increase of Aβ deposition in aged mice, however, a significant decrease in axonal transport rates is observed compared with age-matched control animals (Smith et al. 2007). Not only Aβ, but also tau seem to play an important role in maintaining axonal transport and axonal integrity. Crossing two mouse mutant strains, one of them overexpressing mutated human APP and the other having a knock-out of tau, results in the extensive formation of axonal spheroids and a degeneration of neurites. This is independent of Aβ plaques indicating a crucial function of tau for the maintenance of axonal integrity (Dawson et al. 2010). Furthermore, a local axonal increase in tau concentration has been shown to promote cargo detachment from microtubules, whereas a decrease allows the uninterrupted transport of cargos (Ebneth et al. 1998; Stamer et al. 2002; Vershinin et al. 2007). In spite of this large body of data, the exact interplay between tau and Aβ in the promotion of axonal degeneration is not yet understood in detail.

Taken together, the data provide strong evidence that axonal transport impairment contributes at least to axonal dystrophy, which can, in the end, culminate in axonal degeneration attributable to deficient cargo exchange. A direct visualization of transport impairment, axonal dystrophy and consecutive degeneration by live-imaging methods should certainly strengthen this hypothesis. The finding that axonal transport is gradually impaired in chronic disease and does not immediately result in axonal degeneration offers an especially challenging possibility for putative therapeutic interventions, as the pathology might still be partially reversible. Nevertheless, a process as complex as axonal transport is unlikely to be rescued by one specific intervention. In terms of pharmacological intervention, factors modulating axonal transport further upstream, such as protein aggregation (Aggregation above), calpain-activation (Calcium-triggered events) and the activation of c-Jun-N-terminal kinase (JNK; Kinase activation) might be more promising targets.

#### Kinase activation

Kinases participate in a specific manner in the execution of the axonal destruction program. A few prominent examples shall therefore be discussed in detail here without an attempt to be comprehensive.

The JNKs are known as stress-activated protein kinases because their activities typically increase in response to Various cellular environmental stresses such as osmotic stress, redox stress or irradiation (fro reviews, see Davis 2000; Weston and Davis 2007). Once activated by a stress response or by inflammatory cytokines, they can propagate the signal to induce cellular apoptosis (Kyriakis and Avruch 1996; Ip and Davis 1998). Injury can also activate axonally located JNKs, resulting in axonal transport defects (see Axonal transport) that are associated with the dissociation of heavy-chain kinesin family-5B (KIF5B) protein from tubulin in axons finally leading to increased degeneration (Stagi et al. 2006). For example, activated phospho-JNK has been found to be highly expressed in the lesioned corticospinal tract in a mouse spinal cord injury model and axonal retraction can be inhibited by the application of a pan-JNK inhibitor, which also improves functional recovery (Yoshimura et al. 2011). Vice versa, mutations in the kinesin light-chain-1 are found to result in the activation of JNK, which colocalizes with hyperphosphorylated and accumulated tau in dystrophic axons (Falzone et al. 2009).

As described in detail under Parkinson’s disease, axonal degeneration is also one of the earliest pathological hallmarks of PD (Duda et al. 2006). One of the most frequently employed toxin models for PD, the MPTP mouse model, shows only mild to no neuronal loss in the subacute intoxication paradigm. However, the function of dopaminergic neurons, being most dependent on their axonal structures, is significantly impaired (Fox and Brotchie 2010). Here, the JNK pathway is activated via phosphorylated c-Jun in the striatum and in the substantia nigra; this appears not to be correlated with the loss of neuronal cell bodies but might represent a response to damage/loss of axonal terminals (Willesen et al. 2002), as has been corroborated by the finding that c-Jun is activated in dopaminergic neurons from PD patients. The same authors have shown that, in the acute MPTP mouse model of PD, both JNK2 and JNK3, but not JNK1, are required for MPTP-induced c-Jun activation and dopaminergic cell demise (Hunot et al. 2004). Different JNK isoforms apparently have distinct functions, since JNK2 and JNK3 seem not to participate in retrograde axonal degeneration following 6-hydroxydopamine (6-OHDA) lesion in mice; although the cell soma is protected in JNK2/3 double-null mutants, the axons still degenerate (Ries et al. 2008).

JNK can also be activated by the dual leucine kinase (DLK) in axonal lesion models in *Drosophila* and mice. Indeed, animals deficient for DLK show a delay in Wallerian degeneration after sciatic nerve transection and in a toxin model of vincristine treatment (Miller et al. 2009). Activation and phosphorylation of JNK is delayed in WldS explants suggesting that WldS protein expression regulates JNK activation (Barrientos et al. 2011).

Kinases involved in inhibitory myelin signalling are also involved in the regulation of axonal stability. Myelin-derived inhibitory molecules, such as Nogo (the neurite outgrowth inhibitor), myelin-associated glycoprotein or oligodendrocyte myelin glycoprotein confer their signal through the trimeric NgR/p75/LINGO-1 or NgR/TROY/LINGO-1 receptor complexes (for a review, see McDonald et al. 2011) or, as has been recently identified, via the paired Ig-like receptor B (Atwal et al. 2008). Downstream, Rho-associated kinase (ROCK) integrates the signal from other inhibitory receptors, such as ephrin and semaphorin (Goldberg et al. 2004; Goldshmit et al. 2006). Although this signalling pathway has been mostly studied in paradigms of impaired axonal regeneration, indications have been found that this pathway also participates in triggering axonal degeneration. For example, the blocking of Nogo-A signalling is able to attenuate axonal degeneration in an EAE mouse model (Karnezis et al. 2004).

A recent report has implicated the phosphoinositide 4-kinase 2 alpha (Pi4k2a) in axonal stability. Knockout mice for Pi4k2a develop a progressive neurological disease with tremor, limb weakness, urinary incontinence and premature mortality. Axonal degeneration in the spinal cord has been found to be a major histological correlate of the symptoms, whereas peripheral nerves appear essentially normal. Thus, Pi4k2a signalling appears to be crucial for axonal integrity and therefore *Pi4k2a* has been proposed as a candidate gene for hereditary spastic paraplegia (Simons et al. 2009).

#### Autophagy and the ubiquitin-proteasome system

Degradation of proteins or even organelles occurs in a homeostatic manner via several degradative routes, one of them being autophagy. Autophagy is a collective term and, in this text, we use it to refer to macroautophagy. Because autophagy is a highly conserved and tightly regulated mechanism any disturbance (be it an increase or a decrease) can result in cellular dysfunction.

Following mechanical axonal damage in a crush model of the optic nerve, an increased number of autophagosomes has been observed in the vicinity of the lesion site and this is dependent on calcium influx. Inhibition of autophagy attenuates axonal degeneration in this model (Koch et al. 2010; Knöferle et al. 2010). Autophagy is also induced in degenerating neurites of sympathetic neurons in an axotomy model, as has been substantiated by an accumulation of autophagosomes and increased expression of LC3-II (a 16-kDa protein that localizes to autophagosomal membranes). Treatment with 3-methyladenine (3-MA) and knockdown of autophagy-regulator 7 (Atg7) or Beclin1 are able to counteract the degenerative process partially (Yang et al. 2007). Cerebellar Purkinje cells show an induction of autophagy in axon terminals upon excitotoxicity, with the process appearing to be axon-specific and independent of the cell body (Yue 2007). This has also been elegantly shown in a green fluorescent protein (GFP)-LC3 transgenic mouse strain crossed with the Lurcher mutant strain, in which a mutant glutamate receptor is constitutively activated and causes Purkinje cell degeneration. Here, GFP-LC3-labelled autophagosomes accumulate in axonal dystrophic swellings (Q.J. Wang et al. 2006). Recently, an in-depth examination of four different mouse models of nigrostriatal axon injury has revealed the presence of ultrastructural features of autophagy, such as autophagic vacuoles, in the degenerating neurites. Autophagic puncta are induced proximally and distally of the mechanical lesion site and in a striatal 6-OHDA injection model (Cheng et al. 2011).

Next to autophagy-mediated degradation, the ubiquitin-proteasome system (UPS) has also been shown to be relevant for axonal degeneration. This has been demonstrated by the inhibition of UPS in an optic nerve crush lesion model in which Wallerian degeneration is attenuated by proteasome inhibition. Fragmentation of microtubuli as an early event following crush lesion can be diminished by the application of the proteasome inhibitor MG132 (Zhai et al. 2003). On the other hand, proteasome inhibition by lactacystin causes a dying-back-like degeneration in PC12 cells in vitro (Laser et al. 2003). Thus, a disturbance of proteasome function in one way or another can have detrimental effects, similar to alterations in autophagy.

## Therapeutic targets

### Axonal membrane integrity

Structural lesions, such as the disruption of the axonal membrane and microtubule breakage, are the primary events in trauma-induced axonal degeneration and thus could represent early therapeutic targets. For example, axolemmal pores, which are thought to be responsible for calcium influx in diffuse axonal injury after brain trauma, can be successfully treated in vitro with Poloxamer 188, a triblock polymer, which is able to reseal membrane pores (Kilinc et al. 2009). The hydrophilic polymer polyethylene glycol (PEG) has similar chemical properties with regard to membrane sealing (Shi and Borgens 2000). The application of PEG-decorated silica nanoparticles in a spinal cord lesion model in vivo results in an accumulation of the particles at the lesion site and the recovery of electrical conduction through the lesion site (Cho et al. 2010). Whether such approaches will be applied in future clinical treatments will largely depend on the feasibility of rapid application following injury, targeted delivery to the lesion site and their side-effects profile.

### Inhibitors of calcium and calcium-dependent enzymes

Calcium influx inhibition, for example by L-type calcium channel blockers such as nifedipine or nimodipine, is highly protective in axonal lesion models in vitro. Metal ions substituting for calcium, such as cobalt and manganese, are also able to attenuate axonal degeneration in this model (George et al. 1995). In the optic nerve crush model in vivo, the application of a mixture of amlodipin, amiloride and NBQX (2,3-dihydroxy-6-nitro-7-sulfamoyl-benzo[f]quinoxaline-2,3-dione) prevents the increase of intra-axonal calcium and consecutive axonal destruction. In reverse, the disintegration of the axon is dramatically increased after the addition of the calcium ionophore A23187 to the lesion site (Knöferle et al. 2010). Inhibition of calcium influx by the N-type-specific calcium channel blocker omega-conotoxin GVIA is also axonoprotective in a rat model of autoimmune optic neuritis (Gadjanski et al. 2009).

Inhibitors of calpains have been used as a successful strategy to attenuate axonal damage in several models in vitro and in vivo. For example, dorsal root ganglion axons are protected by calpain inhibitors from axotomy-induced axonal degeneration in vitro (George et al. 1995) and from acute axonal degeneration in the spinal cord in vivo (Kerschensteiner et al. 2005). Calpain inhibition has been shown to be protective in models of traumatic brain injury; it successfully prevents axonal beading after shear stress (Kilinc et al. 2009) and is also protective in a model of anoxic axonal damage (Jiang and Stys 2000). After fluid percussion injury, the application of the calpain inhibitor MDL-28170 exhibits protective effects against axonal destruction. As might be expected from the disease mechanism, earlier administration of the drug shows a better axonoprotection than application at a later time point with the best results being achieved when MDL-28170 is administered before lesion (Ai et al. 2007). Interestingly, both studies have demonstrated only partial functional recovery, suggesting that calpain is not the only calcium-dependent downstream target and that other evasive pathways might have been initiated at the same time. Thus, even calpain inhibition may lie too far downstream in terms of functional rescue.

Although the inhibition of calcium influx or intra-axonal calcium release appears to be one of the most potent approaches in the prevention of further degeneration, the kinetics of calcium flux render calcium an unfavourable target for therapeutic interventions in acute lesions. Calcium influx in traumatic lesions usually occurs so fast that a timely intervention appears unrealistic (Knöferle et al. 2010). However, the targeting of calcium could be beneficial in chronic disorders, which have a much slower progression, and this is supported, for example, by the EAE model data. Indeed, riluzole, the only drug licensed for the treatment of ALS, acts as an NMDA receptor antagonist and thus regulates calcium influx to the cell, a possible explanation for its beneficial effects in this motoneuron disorder (Van Damme et al. 2005). Interestingly, recent data from a retrospective Danish population-based study employing logistic regression analysis suggest that subjects prescribed dihydropyridines are less likely to develop PD; this indicates a possible neuroprotective role for these centrally acting L-type calcium channel blockers (Ritz et al. 2010). Evidence is thus accumulating that calcium can also be a target in chronic neurodegenerative disorders, a finding that is of special interest, because many calcium channel blockers have well-known pharmacokinetics in humans and are already widely used in clinical practice.

### WldS/NMNAT

The WldS mutation has been shown to be neuroprotective in several models of chronic degenerative CNS disease such as PD, glaucoma, MS, Charcot-Marie-Tooth disease 1A and axonal dystrophy (Mi et al. 2005; Kaneko et al. 2006; Hasbani and O’Malley 2006; Howell et al. 2007; Wilbrey et al. 2008; Beirowski et al. 2008; Meyer zu Horste et al. 2011). In a recent study evaluating various models of PD, the WldS mutation is protective in anterograde but not retrograde degeneration suggesting that both processes involve different molecular mechanisms (Cheng and Burke 2010).

Further analysis of the biologically effective mechanism of WldS has revealed that the enzymatic activity of NMNAT1 together with the VCP-binding site of UBE4b are required to achieve the full neuroprotective effect comparable to WldS-expression (Avery et al. 2009; Conforti et al. 2009; Yahata et al. 2009). However, in *Drosophila*, the overexpression of NMNAT3, an isoform of NMNAT that localizes primarily to the cytosol and mitochondria, provides axon protection indistinguishable from that of WldS. This shows not only the potential therapeutic value of NMNAT3 in treating degenerative disease, but also the importance of the subcellular localization of the active substrate. Complete loss of NMNAT causes severe axonal degeneration in *Drosophila* sensory neurons supporting its essential role in the maintenance of axonal and dendritic integrity (Wen et al. 2011). Consistently, axonally targeted NMNAT1 is highly axonoprotective in a mouse model of Wallerian degeneration, although the precise mechanism of action is still under debate (Babetto et al. 2010).

### Kinases

The diversity among kinases is reflected in the plethora of pharmacological kinase inhibitors available. Here, we focus on a few examples, namely inhibitors of ROCK, JNK, GSK-3 and IkappaB (IκB), for which particular axonoprotective effects have been described.

Interference with ROCK function has recently been shown to be beneficial in various disease models as it is able to confer neuroprotection and promote axonal regeneration (Lingor et al. 2008; Tönges et al. 2011). However, evidence has also been obtained that ROCK inhibition protects from axonal degeneration. When axons are mechanically severed, they retract because of increased actomyosin contractility. This is based on the regulation of myosin II by myosin light-chain kinase (MLCK), which directly phosphorylates myosin regulatory light chains and activates myosin motor activity. Activated ROCK increases the phosphorylation of myosin regulatory light chains and additionally inhibits myosin light-chain phosphatase by phosphorylation. It possibly even directly phosphorylates the myosin regulatory light chains themselves (Luo 2000). In an in vitro-approach, the application of the pharmacological ROCK inhibitor Y-27632 to severed chicken retinal axons has been shown to inhibit myosin light-chain phosphorylation and thereby significantly decreases the axonal retraction distance (Gallo 2004).

In a mouse model of EAE, both the parenteral and oral administration of the ROCK inhibitor fasudil prevents the development of EAE induced by proteolipid protein. In addition to a reduction of the specific proliferation of lymphocytes, a downregulation of interleukin (IL)-17 and a marked decrease of the interferon-gamma/IL-4 ratio, CNS demyelination and acute axonal transections are robustly attenuated (Sun et al. 2006). This protection from axonal degeneration has been confirmed by a study in a rat model of experimental autoimmune neuritis (EAN)in which ROCK inhibition can even reduce EAN severity when administered after disease onset. Again, inflammatory cell infiltration is markedly decreased and the secretion of inflammatory cytokines is reduced (Pineda et al. 2011). The mechanisms of action of ROCK inhibition with regard to the attenuation of axonal degeneration thus seem to be manifold and at least partially mediated via the modulation of inflammatory processes and interference with cytoskeleton motility.

JNKs have been proposed as therapeutic targets for several neurodegenerative disorders. However, in addition to their neuroprotective action, experimental evidence now suggests that JNK inhibition protects degenerating axons. For example, the application of SP600125, a pan-JNK inhibitor, attenuates axonal retraction after spinal cord lesion and improves the motor outcome (Yoshimura et al. 2011). A shortcoming of JNK inhibition as a therapeutic approach might be the broad range of JNK-mediated effects, which result from the association of JNKs with other proteins in a variety of signalosomes. Here, a selective targeting of JNKs will not be sufficient but will require peptides that block molecular domains of such JNK signalling complexes (Waetzig and Herdegen 2005).

Finally, in a recent large scale screen of 480 bioactive compounds, inhibitors of GSK-3 and IκB kinase have been found to delay the fragmentation of severed axons markedly in vitro. This has been verified by short-hairpin- RNA-mediated knock-down of these target genes (Gerdts et al. 2011). Although this data has to be verified in vivo, these kinases might represent novel pharmacological targets in axonal degeneration.

From a therapeutic perspective, kinase inhibitors are promising substances and several drugs have already been licensed for human use, mostly in the field of anti-cancer therapy. Because kinase inhibitors are usually not completely specific, target selectivity will be a major criterion in the selection process when it comes to therapeutic considerations.

### Autophagy

The therapeutic range of pharmacological approaches regulating autophagy appears to be narrow because a basal level of autophagy is essential for all cells, whereas slight shifts of this equilibrium in either direction might cause pathology (for excellent reviews, see Chu et al. 2009; Jaeger and Wyss-Coray 2009).

For acute axonal degeneration in the rat optic nerve in vivo, we have been able to show a clear axon stabilizing effect of autophagy inhibition with 3-MA, suggesting that autophagy inhibition in the early phase after a traumatic nerve injury might be beneficial (Knöferle et al. 2010). This protective effect on neurite integrity has also been found by several other groups in other in vitro models: the inhibition of autophagy by small molecules or siRNA results in attenuated neurite degeneration and increases cell viability in a growth-factor-deprivation model in mouse superior cervical ganglion neurons (Yang et al. 2007). In agreement, Atg7 knockdown also partially protects neurites from degeneration in the first 12 h following axonal transection in the same cell culture model. Similarly, autophagy inhibition in Atg7 knock-out mice results in protection from neuron death and axonal degeneration in a hypoxic-ischaemic injury model (Koike et al. 2008).

On the other hand, autophagy seems to be neuroprotective under certain conditions depending on the phase and form of axonal degeneration. For instance, in an in vivo model of mouse traumatic brain injury, treatment with the autophagy-inducing drug rapamycin significantly improves functional recovery (Erlich et al. 2007) and similar results have been obtained by other groups in related paradigms (Egami et al. 2005; Sadasivan et al. 2008).

In chronic neurodegenerative disease models, therapeutic modulation of autophagy in either direction has been shown to be beneficial according to the specific model. For example, activation of autophagy by the mTOR (mammalian target of rapamycin) inhibitor rapamycin attenuates the accumulation of mutant huntingtin and is neuroprotective in a *Drosophila* model of Huntington’s disease (Ravikumar et al. 2004). Conversely, application of the autophagy inhibitor 3-MA or knock-down of autophagy-regulators Atg5 or Atg12 is neuroprotective in the MPP^+^ and alpha-synuclein (A53T) cell culture models of PD (Wong et al. 2011).

The regulation of autophagy in a therapeutic manner will largely depend on increasing knowledge of the regulation of this pathway in specific pathologies. Part of this improved understanding must be a more detailed temporal resolution. Autophagic flux might be increased at one point after lesion but may return to normal levels or be inhibited at another. A unidirectional approach, which does not take into account the kinetics of autophagic activity, might therefore result in unwanted detrimental effects by interference with this conserved physiological mechanism.

### Aggregation

The search for effective compounds or strategies to prevent protein aggregation is one of the hottest topics in current neurodegeneration research. Because of primary axonal damage in the course of aggregopathies, this approach holds the promise of achieving true neuroprotective and possibly neurorestorative effects. One therapeutic approach that has been tested extensively in AD mouse models is the immunization of animals with antibodies against Aβ. In an APP mutant mouse model (APPswe/PS1dE9), the application of an anti-Aβ antibody has not only resulted in a modest reduction of Aβ in the brain, but can also significantly attenuate axonal degeneration resulting in higher axon densities in the cortex and hippocampus of the treated mutant mice (Liu et al. 2011). Targeting the aggregation of tau seems similarly promising. Tau is a microtubule-associated protein and both the loss and the toxic gain of function plus the hyperphosphorylation and aggregation of tau have been prime examples for aggregation-related axonal dystrophy and degeneration, for example in AD. Oligomeric forms of tau have been shown to impair fast axonal transport in vitro. This effect can be attenuated by the addition of heat shock protein 70 to oligomeric and fibrillar tau (Patterson et al. 2011). The use of drugs previously tested in human trials and licensed for other pharmacological purposes is especially auspicious. In this context, immunophilin ligands such as FK506 (an immunosuppressant that has been in clinical use for more than 15 years) have recently showed remarkable effects. FK506-binding protein (FKBP) family members have previously been demonstrated to accelerate the aggregation of alpha-synuclein in vitro. In a cell culture model of synucleinopathy, FK506 has been shown to inhibit alpha-synuclein aggregation and neuronal cell death. Similarly, aggregate formation is reduced and cell viability is improved after oral administration of FK506 in an AAV-based mouse model of PD in vivo (Gerard et al. 2010). Nevertheless, approaches involving interference with aggregation will have to be carefully tuned in order to avoid converse effects. An increase of toxic oligomeric species at the cost of less fibril formation might result in exactly the opposite outcome from that desired, as has been shown for the disaccharide trehalose; although Aβ40 aggregation can be inhibited by trehalose, the same treatment prevents only the formation of fibrillar forms of Aβ42, toxic oligomeres being still present and the toxicity of Aβ42 not being attenuated (Liu et al. 2005).

### Growth factors

Trophic factors exert an influence on growth, differentiation and survival of neuronal populations during development and in the adult. Because of their pleiotrophic effects, they have not only been suggested as therapeutic agents for a number of neurological disorders, but have already been tested in human trials (e.g. Gill et al. 2003). Here, we will review only a few prominent examples that have shown protective effects in terms of axonal degeneration.

Viral vector-mediated overexpression of the neurotrophic factor glial-cell-line derived neurotrophic factor (GDNF) in the substantia nigra is able to improve axonal stability and somatic survival of dopaminergic neurons in the 6-OHDA model of PD. Thus, 6-OHDA-mediated axonal dying-back pathology can be rescued, at least partially, by GDNF (Mandel 1997). Kirik et al. (2000) have shown that GDNF application to the striatum is required in this model in order to protect the projecting nigrostriatal fibres. Thus, the location of growth factor application needs to be considered from a technical point of view. On the other hand, GDNF is not sufficiently protective in a model of alpha-synuclein-induced axonal degeneration and dopaminergic terminals are equally unprotected by lentiviral GDNF expression. Corresponding to this finding, the expression of GDNF also does not prevent the aggregation of alpha-synuclein in dopaminergic terminals (Decressac et al. 2011). Lentiviral vector application of GDNF is furthermore not protective against axonal degeneration induced by the overexpression of the human pathogenically mutated A30P alpha-synuclein (Lo Bianco et al. 2004). All this makes clear that a growth factor such as GDNF acts only on parts of the degenerative cascade and a therapeutic effect in humans will be limited if major pathomechanisms, such as aggregation, are not sufficiently addressed. Axonal transport deficits might also be responsible for the limited effect of growth factor therapy in human PD patients, because the trophic factors are not sufficiently transported to the cell soma. For example, striatal delivery of AAV-neurturin will require much longer retrograde transport in humans than in experimental models involving smaller mammals, i.e. non-human primates or rodents (Bartus et al. 2011).

In an axotomy model, local application of ciliary neurotrophic factor is able to prevent the degeneration of rat facial motoneurons (Sendtner et al. 1990) and a more recent report presents data on ciliary neurotrophic factor (CNTF)-mediated protection in axonal pruning of motoneurons in the SOD1(G93A) mouse model (Pun et al. 2006). Here, the stalling of synaptic vesicles has been identified as an early sign of axonal dysfunction and the application of CNTF is able to reverse this pathology, whereas GDNF does not. Axonal die-back induced by axotomy of the optic nerve can be inhibited by the local application of brain-derived neurotrophic factor and also by neurotrophin-3 and CNTF (Weibel et al. 1995).

Erythropoietin (EPO) is a multifunctional cytokine that not only controls erythropoiesis, but has also been attributed neuroprotective properties (e.g. Grunfeld et al. 2007; Xue et al. 2010; Reitmeir et al. 2011). Several studies in a model of peripheral neuropathy (acrylamide-mediated axonal injury) have demonstrated that EPO is the functional mediator of NO-induced axonal protection and can be released by Schwann cells upon injury. EPO signalling in this model is dependent on the activation of hypoxia-inducible factor-1α (Keswani et al. 2004, 2011).

## Concluding remarks

The molecular machinery of axonal degeneration still requires a much better understanding and this can be partially achieved through in vivo visualization of the implicated processes. Here, life-imaging techniques (Misgeld and Kerschensteiner 2006) and MRI methods (Ge 2006; Fox et al. 2011; Kim et al. 2011) might yield more information and further insights can be expected from the evolution of these methods (Westphal et al. 2008). Because of the multitude of aetiologies and molecular mechanisms involved in axonal degeneration, therapeutic approaches aimed at prevention or restitution will probably require multiple strategies. Intriguingly, in spite of the pathological diversity of axonal degeneration, some keystones of the degenerative cascade and thus some key targets appear to be identical. The regulation of axonal calcium levels, for example, is of major importance for acute and chronic degenerative paradigms. On the other hand, the kinetics of intra-axonal calcium levels represents a challenge when it comes to a therapeutic application. Alterations of homeostatic mechanisms, such as autophagy, are equally efficient in modulating axonal stability but therapeutic intervention appears to depend on a narrow time window, outside of which any interference with such essential cellular processes might lead to detrimental instead of beneficial effects. Questions of administration routes, tissue specific targeting, timing and duration are not discussed in this review but will have to be considered with respect to the design of axonoprotective therapies.
